# Bacteriophages isolated from Lake Michigan demonstrate broad host-range across several bacterial phyla

**DOI:** 10.1186/s12985-015-0395-0

**Published:** 2015-10-09

**Authors:** Kema Malki, Alex Kula, Katherine Bruder, Emily Sible, Thomas Hatzopoulos, Stephanie Steidel, Siobhan C. Watkins, Catherine Putonti

**Affiliations:** Department of Biology, Loyola University Chicago, Chicago, IL 60660 USA; Department of Computer Science, Loyola University Chicago, Chicago, IL 60611 USA; Bioinformatics Program, Loyola University Chicago, Chicago, IL 60660 USA

**Keywords:** Bacteriophage, Broad host-range, Freshwater, Lake Michigan

## Abstract

**Background:**

The study of bacteriophages continues to generate key information about microbial interactions in the environment. Many phenotypic characteristics of bacteriophages cannot be examined by sequencing alone, further highlighting the necessity for isolation and examination of phages from environmental samples. While much of our current knowledge base has been generated by the study of marine phages, freshwater viruses are understudied in comparison. Our group has previously conducted metagenomics-based studies samples collected from Lake Michigan - the data presented in this study relate to four phages that were extracted from the same samples.

**Findings:**

Four phages were extracted from Lake Michigan on the same bacterial host, exhibiting similar morphological characteristics as shown under transmission electron microscopy. Growth characteristics of the phages were unique to each isolate. Each phage demonstrated a host-range spanning several phyla of bacteria – to date, such a broad host-range is yet to be reported. Genomic data reveals genomes of a similar size, and close similarities between the Lake Michigan phages and the Pseudomonas phage PB1, however, the majority of annotated genes present were ORFans and little insight was offered into mechanisms for host-range.

**Conclusions:**

The phages isolated from Lake Michigan are capable of infecting several bacterial phyla, and demonstrate varied phenotypic characteristics despite similarities in host preference, and at the genomic level. We propose that such a broad host-range is likely related to the oligotrophic nature of Lake Michigan, and the competitive benefit that this characteristic may lend to phages in nature.

## Findings

In the last thirty years, studies of bacteriophages in the environment continue to demonstrate the importance of phages as drivers of bacterial mortality and diversity. Most of our knowledge with regard to phage diversity, in a wide range of niches, has come from metagenomic surveys [1, 2]. However, the majority of virally-related sequences generated by such surveys exhibit no amino acid similarity with annotated genes [3]. As technology improves, it has become clear that a severe paucity of genomic and phenotypic information exists for isolated environmental phages: this lack of information severely limits what can be achieved through deep-sequencing techniques alone. The high percentage of ORFans (open reading frames with no known match to coding DNA) identified in metagenomic studies [4] can be improved upon by the isolation and examination of phages directly from the environment. Comparatively, cultivation of phages allowing for the sequencing of clonal genomes: consequently, this improves the content of available databases. It also allows for the examination of physical features under high powered microscopy, such as transmission electron microscopy (TEM) and atomic force microscopy (AFM) [5, 6].

Current understanding of environmental phages has largely come from those which are aquatic in origin, however, freshwater phages are understudied in comparison to their marine counterpart and isolated phages are often cyanophages [7–9]. To date, few studies exist pertaining to specific characterization of freshwater phages that do not infect cyanobacteria [10], rendering them particularly under-represented in databases such as RefSeq. Representation of freshwater phages is of considerable importance when one considers the close proximity these environs often have to human activity: for example, the potential for anthropogenically-mediated pollution (e.g., sewage outfall), and, when considering Lake Michigan in particular, the use of freshwater as a source of drinking water and recreation. Furthermore, the heterogeneous nature of freshwater environments with regard to aspects such as water chemistry, and, crucially, bacterial diversity, suggest that viral diversity is likely to be greater than that observed in marine sample sets [11]. While there is evidence to suggest some evolutionary relationship between marine and freshwater phages [12, 13], conflicting results have also been reported [14] and a considerable degree of divergence is to be expected between the two groups.

Assessment of phage diversity in the environment should also take into account factors of phage lifestyle (e.g., lysis and lysogeny) and phage host-range. Regarding host-range, previous studies have assigned classification as a ‘generalist’ when a phage demonstrates capacity to infect more than one species of a bacterial genus [15], some restricting the definition further to include strains of a particular species [16, 17]. This is a difficult issue to address when considering concepts of bacterial species [18]. Here, we examine phages which are able to infect different bacterial phyla. In some instances, isolated phages that have been classified as generalists have been shown to demonstrate defined characteristics, such as reduction in infection efficiency compared to specialists [19]. This suggests that qualification as a true generalist may rely on more than just host-range. This notwithstanding, here we present four phages which are able to infect laboratory type strains, environmental and co-isolated environmental bacterial strains belonging to the phyla Proteobacteria, Actinobacteria and Bacteroidetes. Broad host-range is congruent with existing theory suggesting that generalism is of benefit of phages inhabiting an oligotrophic environment, such as Lake Michigan.

During this investigation, all phages were propagated on bacteria grown on/in lysogeny broth agar/lysogeny broth, at 37 °C. Four phages were isolated from the nearshore waters of Lake Michigan on *E. coli* (ATCC 8739), from the same samples used by the authors previously to investigate a metaviromic dataset generated from Lake Michigan [20]. Plaques originally isolated on *E.coli* were removed as single plugs and sequentially purified to generate clonal phage cultures. Host-range testing demonstrated lysis of laboratory strain *P. aeruginosa* (ATCC 15692), four strains of *P. aeruginosa* co-isolated from the same Lake Michigan samples, as well as strains of *Arthrobacter, Chryseobacterium* and *Microbacterium* (identified to the genus level via amplification and sequencing of the 16S rRNA gene) (Table 1). The phages were not able to infect *E.coli* K12, *Achromobacter* sp., *Salmonella enterica*, *Shigella boydii* or *S. flexneri*. Growth characteristics of the phages were assessed via one-step growth curve experiments on *P.aeruginosa* (ATCC 15692), performed at an m.o.i. of 0.1 with bacterial cells maintained at late exponential phase. Each isolate demonstrated differences in length of latent period (10–120 mins) and generation time (60–100 mins). Burst size for all four isolates remained within an order of magnitude.

**Table 1:** Phage characterization as determined from host-range assays and one-step growth curves

	ɸFenriz	ɸHabibi	ɸMoody	ɸVader
Infection:				
*P. aeruginosa* (ATCC 15692)	+	+	+	+
*E. coli* (ATCC 8739)	+	+	+	+
*Arthrobacter* sp.	+	+	+	+
*Chryseobacterium* sp.	+	+	+	+
*Microbacterium* sp.	+	+	+	+
Latent period (mins)	120	30	90	10
Generation time (mins)	180	90	150	60
Burst size (PFU/ml)	6.13×10^9^	1.52×10^9^	2.67×10^9^	3×10^9^

**Fig. 1:**
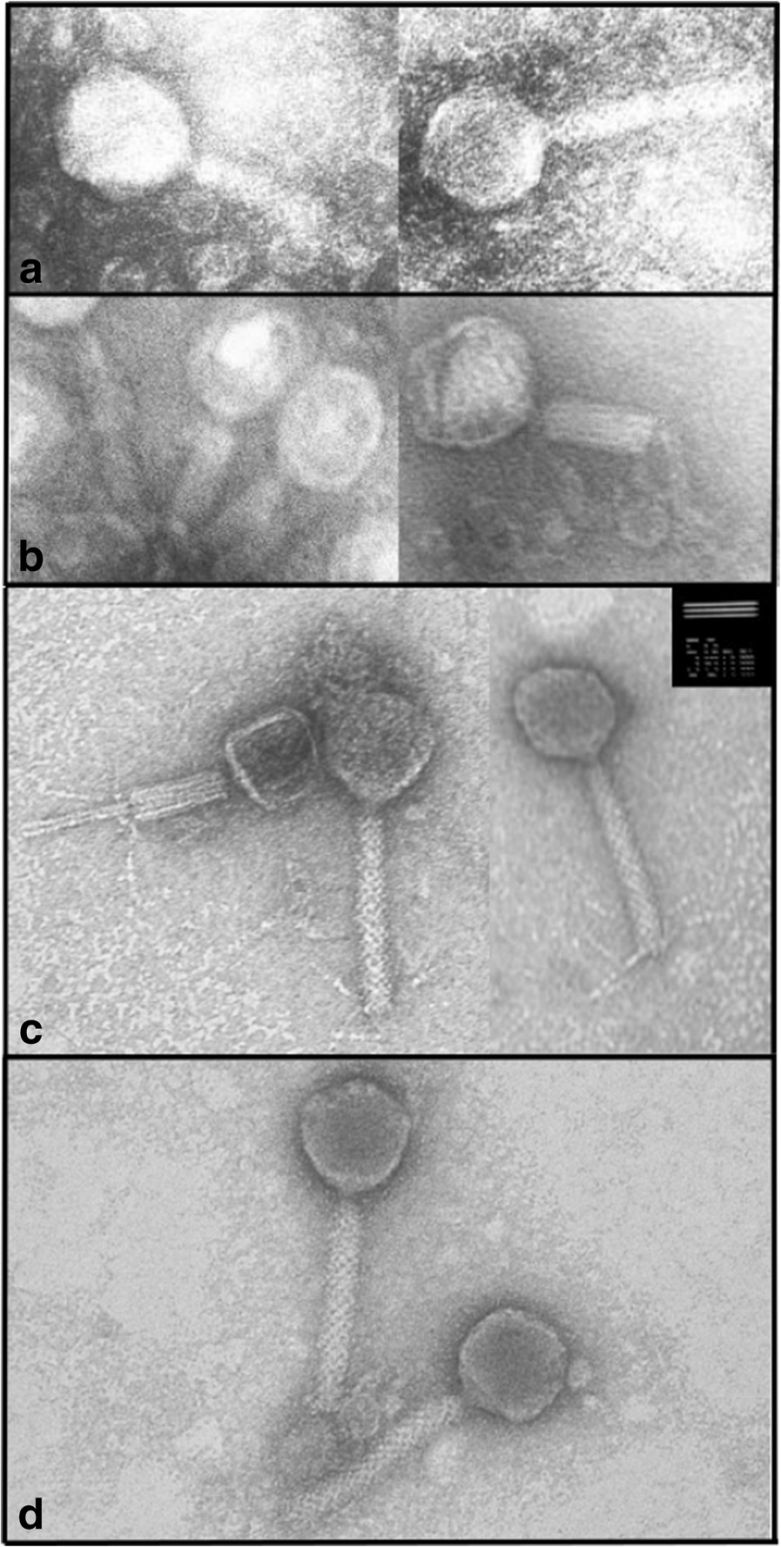
TEM micrographs of phages ɸFenriz (**a**), ɸHabibi (**b**), ɸMoody (**c**) and ɸVader (**d**). Scale bar represents 50 nm

Transmission electron micrographs of the four phages (Fig. 1), all of which demonstrated morphological characteristics typical of *Myoviridae*, showed contractile tails (retracted tails were particularly evident in panel C) approximately 150 nm in length, and capsids at approximately 60 nm diameter, apart from ɸMoody (Fig. 1, panel c) which was slightly smaller at 50 nm.

Infected cultures were saturated with chloroform, passed through a 0.22 μm filter and concentrated via tangential flow filtration. The final fraction was subject to DNase I treatment, and DNA extracted using the UltraClean® Microbial DNA Isolation Kit (MO BIO Laboratories, Carlsbad, CA). Library construction and sequencing was conducted at the University of Texas Medical Branch (Galveston, TX). Each of the five isolates was multiplexed and sequenced using the Illumina MiSeq platform via the MiSeq Reagent Kit v2 (500 cycle), producing paired-end reads each 250 nucleotides in length.

**Fig. 2:**
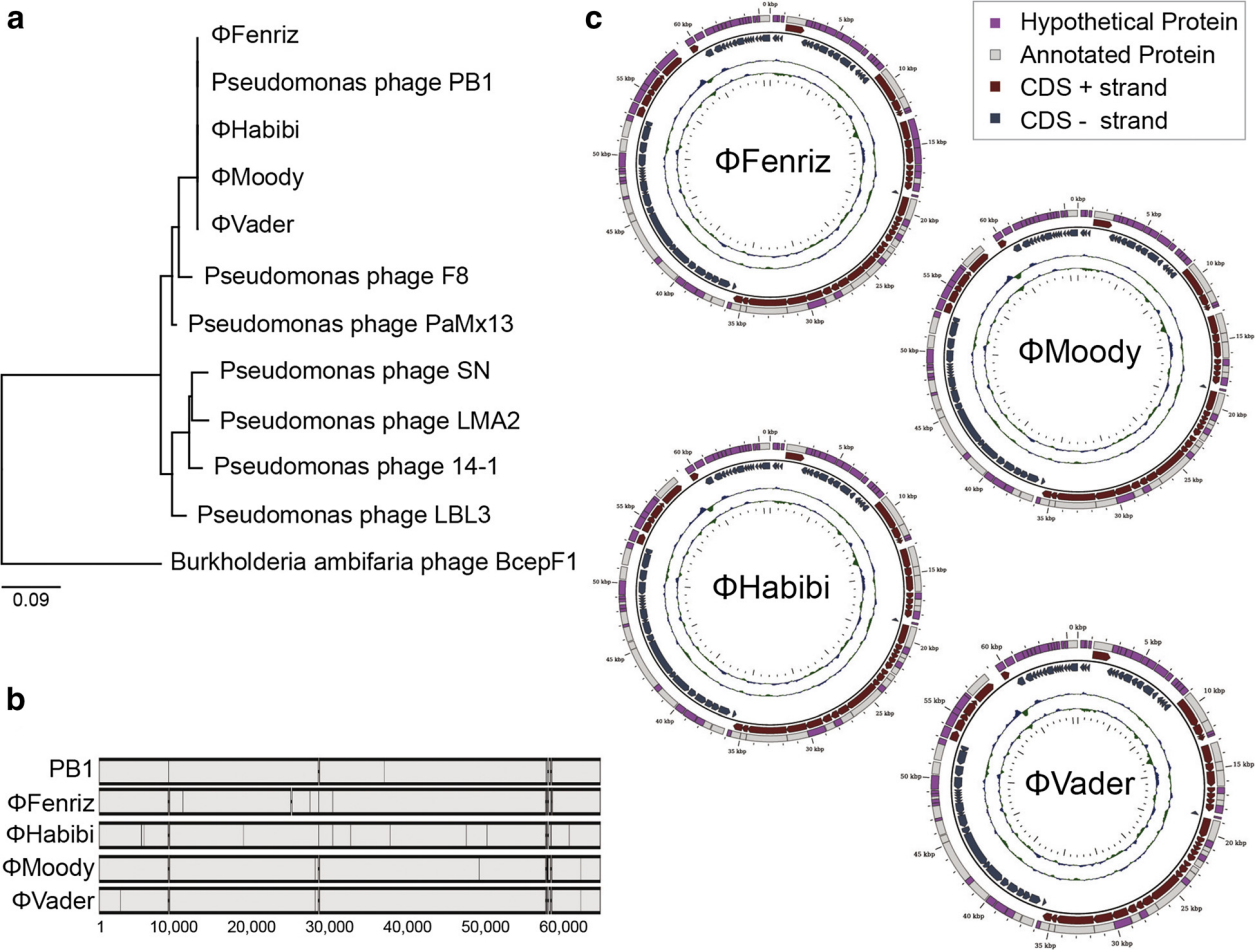
Characterization of the four Lake Michigan isolates at the genomic level. Lake Michigan isolates were compared phylogenetically to other PB1-like viruses with a neighbor-joining tree (**a**), phage genomes were also directly compared to PB1 (**b**). Finally, genomes of the phages isolated as part of this study were closed and annotated (**c**)

The genomes for the four Lake Michigan isolates were assembled and annotated using Velvet [21] (in Geneious, running VelvetOptimiser for selection of k), PhagePhisher (Hatzopoulos T, Watkins SC, Putonti C. PhagePhisher: a pipeline for the discovery of covert viral sequences in complex genomic datasets. submitted), and RAST [22]. While de novo assembly of ɸVader and ɸHabibi produced a single contig, four and five contigs for ɸMoody and ɸFenriz, were identified and subsequently closed via PCR. The data supporting the results of this article were deposited in NCBI (Genbank Accession numbers KT254130, KT254131, KT254132, and KT254133). Fundamentally, the four phages were similar in terms of genome length, GC content and number of predicted genes (Table 2). With regard to existing data, all four showed greatest similarity to Pseudomonas phage PB1 [23] and fell out into a distinct clade when compared to other PB1-like viruses (Fig. 2, panel a). Sequence identity to PB1 for each of the four isolates was 99 %, including mismatches as well as regions of homology between the Lake Michigan isolates absent from the PB1 genome (Fig. 2, panel b).ɸFenriz uniquely consisted of two frame shift mutations, which disturbed the coding regions of a putative minor head protein and putative structural protein. Between the isolates, variation was limited to five non-synonymous mutations at most: i.e., between ɸHabibi and ɸMoody, and ɸHabibi and ɸVader. ɸMoody and ɸVader differed by two non-synonymous mutations (Fig. 2, panel c).

**Table 2:** Genomic characteristics of isolated phages

Phage	Genome length	%GC	Predicted genes
ɸFenriz	65,760	54.94	91
ɸHabibi	65,763	54.91	90
ɸMoody	65,908	54.90	90
ɸVader	65,762	54.93	90

The four phages examined in this study represent a group demonstrating a breadth of host-range capacity that has not been reported before. Host-range was assessed on ATCC strains of bacteria as well as those isolated from the environment recently. No conclusions with regard to host-range were offered from genomic level study of the phages, and in at least one of the isolated phages, the potential for lysogeny was observed (not shown) – however, again, no evidence was apparent for the repressor systems that directly mediate lysogeny in other phages. It is likely that synonymous mutations may be responsible for the breadth of phenotypic variations observed in the phages characterized here.

Clearly, the hosts identified here are very different from one another and likely the mode of infection for each candidate host is also very different. The determination of these mechanisms is now an important aspect of describing how broad host-range capacity is possible. That phages isolated from an oligotrophic lake would be able to infect across such a broad spectrum makes sense, fundamentally – in a system where host diversity may be high, but abundance low, this is a strong competitive strategy. That these phages demonstrate a host-range which extends beyond a single bacterial phyla also has clear implications for assigning taxonomical classification to viruses during metagenomic studies. This further emphasizes the importance of culture-based examinations which run parallel to those which are molecular-based.
